# Drawing the line between commensal and pathogenic *Gardnerella vaginalis *through genome analysis and virulence studies

**DOI:** 10.1186/1471-2164-11-375

**Published:** 2010-06-11

**Authors:** Michael D Harwich, Joao M Alves, Gregory A Buck, Jerome F Strauss, Jennifer L Patterson, Aminat T Oki, Philippe H Girerd, Kimberly K Jefferson

**Affiliations:** 1 Department of Microbiology and Immunology, Medical College of Virginia Campus of Virginia Commonwealth University, 1101 E. Marshall Street - PO Box 980678, Richmond, VA 23298 USA; 2 Department of Obstetrics and Gynecology, Medical College of Virginia Campus of Virginia Commonwealth University, 1101 East Marshall Street - P.O. Box 980034, Richmond, VA 23298 USA

## Abstract

**Background:**

Worldwide, bacterial vaginosis (BV) is the most common vaginal disorder. It is associated with risk for preterm birth and HIV infection. The etiology of the condition has been debated for nearly half a century and the lack of knowledge about its cause and progression has stymied efforts to improve therapy and prevention. *Gardnerella vaginalis *was originally identified as the causative agent, but subsequent findings that it is commonly isolated from seemingly healthy women cast doubt on this claim. Recent studies shedding light on the virulence properties of *G. vaginalis*, however, have drawn the species back into the spotlight.

**Results:**

In this study, we sequenced the genomes of a strain of *G. vaginalis *from a healthy woman, and one from a woman with bacterial vaginosis. Comparative analysis of the genomes revealed significant divergence and *in vitro *studies indicated disparities in the virulence potential of the two strains. The commensal isolate exhibited reduced cytotoxicity and yet the cytolysin proteins encoded by the two strains were nearly identical, differing at a single amino acid, and were transcribed at similar levels. The BV-associated strain encoded a different variant of a biofilm associated protein gene and demonstrated greater adherence, aggregation, and biofilm formation. Using filters with different pore sizes, we found that direct contact between the bacteria and epithelial cells is required for cytotoxicity.

**Conclusions:**

The results indicated that contact is required for cytotoxicity and suggested that reduced cytotoxicity in the commensal isolate could be due to impaired adherence. This study outlines two distinct genotypic variants of *G. vaginalis*, one apparently commensal and one pathogenic, and presents evidence for disparate virulence potentials.

## Background

*G. vaginalis *has had a checkered taxonomic beginning. It was originally isolated by Leopold [1] and later associated with the vaginal disorder now referred to as bacterial vaginosis and named *Haemophilus vaginalis *by Gardner and Dukes [2,3]. Subsequently, metabolic requirements and gram staining led to its reclassification within the genus *Corynebacterium*. Greenwood and Pickett suggested that the organism did not belong in this genus either and that it be placed in its own genus, named after its discoverer [4]; a contention later supported by DNA-DNA hybridization [5]. *Gardnerella *is in the Family *Bifidobacteriaceae *and is most closely related to species in the Genus *Bifidobacterium*. The cells are small, nonmotile pleomorphic rods, which may be piliated. The length of the rods and gram staining vary depending on the growth medium [6]. Electron microscopy and the lack of lipopolysaccharide production demonstrates that the cell wall is gram-positive, although the peptidoglycan layer can be thinner than many gram-positive organisms, resulting in negative gram staining [7]. *G. vaginalis *is a fastidious organism and requires complex medium for growth. Studies using metabolic methods of identification indicate that it is catalase-negative, exhibits α-glucosidase activity, starch hydrolysis, hippurate hydrolysis, acid phosphatase activity, but lacks gelatin and esculin hydrolysis, and salt tolerance [6]. *G. vaginalis *is anaerobic and can utilize the carbohydrates dextrin, fructose, glucose, maltose, ribose, starch, and sucrose through fermentation. Some strains ferment mannose, galactose, and sucrose, and a few strains ferment xylose and trehalose but it does not ferment mannitol, raffinose, rhamnose, or sorbitol [6].

Despite the fact that bacterial vaginosis (BV) is the leading vaginal disorder globally, very little is known about its etiology or pathogenesis. It fails to conform to any of Koch's postulates; it is not associated with a single bacterial species, no single species has ever been found to reliably elicit the disorder in healthy women, and the adaptation of the bacterial species involved to life in the human host has precluded the development of a useful animal model. Gardner and Dukes identified *G. vaginalis *as the etiologic agent but findings that pure cultures did not always cause BV drew this allegation into question [3,8]. Subsequent studies analyzed the role of additional species such as *Atopobium vaginae *and *Mycoplasma hominis*, but efforts have yet to unequivocally establish the role of a single species [9,10]. Recent studies of the virulence properties of *G. vaginalis *have revealed its ability to adhere avidly to and establish a tenacious biofilm on the vaginal epithelium in women with BV and have characterized the molecular basis for its cytotoxicity [11,12]. These virulence studies along with findings that *G. vaginalis *is the only species that can be detected in the vast majority of cases of BV and increasing reports of extravaginal infections in which it is the only species isolated, such as bacteremia and osteomyelitis, have drawn this species back into the spotlight and resurrected the notion of its pathogenic potential [13-16].

The role of *G. vaginalis *in BV has also been disputed because the organism can inhabit the genital tract of healthy women [17,18]. However, the numbers of *G. vaginalis *on the vaginal epithelium of healthy women are several logs lower than the numbers found in women with BV [11,18,19]. In addition, it has been reported recently that the biotypes of *G. vaginalis *isolated from healthy women differ from those isolated from women with BV [20]. The lack of genetic characterization of this organism leaves open the possibility that distinct pathogenic and non-pathogenic strains or even subspecies exist. The Human Microbiome Project, an effort supported by the NIH, will soon produce a tremendous amount of genetic information about the bacteria associated with BV and efforts are underway to sequence strains of *G. vaginalis*, *A. vaginae*, and *Mobiluncus*. In an effort to better understand the physiology and pathogenic potential of *G. vaginalis*, we performed whole genome sequence analysis of an isolate from a case of BV (*G. vaginalis *strain AMD), and to determine whether isolates may vary in their pathogenic potential, we also performed whole genome sequence analysis of an isolate from a healthy woman (*G. vaginalis *strain 5-1). We analyzed the virulence potential of these two strains using a series of *in vitro *assays to quantify their cytotoxic activities, their ability to adhere to cervical epithelial cells, and their capacity for biofilm formation.

## Results

### Whole genome sequence analysis

Pyrosequencing yielded high coverages of the BV-associated *G. vaginalis *strain, 5-1 (accession number: ADAM00000000) and the healthy subject isolate, AMD (accession number: ADAN00000000) genomes (~175X and ~130X, respectively). The data for both genomes assembled into approximately 20 contigs greater than 500 bp. Both genomes were estimated at approximately 1.65 Mb. In a MUMmer alignment of the genomic sequences, the minimum percent identity between the two strains was 76.66% and the maximum was 99.07%, with an overall average of 93.62%. GBrowse sites were designed for strains 5-1 and AMD http://www.gardnerella.mic.vcu.edu. Overall GC content figures were 41.95% (5-1) and 42.08% (AMD), and the numbers of protein coding genes predicted were 1,340 (5-1) and 1,318 (AMD). There was only one complete assembled copy of the ribosomal RNA gene cluster, suggesting that all copies are identical or close to it. Examination of contig boundaries suggests that there are at least two and probably three or four copies of the rRNA gene cluster. The number of predicted tRNAs was identical in the two strains: 45.

### Metabolic pathways in the two strains

Analysis of the metabolic potential yielded interesting insights into the basis for the complex growth requirements of this fastidious organism [21]. Metabolic pathways for amino acid synthesis are absent, apart from a few possible short conversions including fumarate to L-aspartate. Pathways for O-glycan, N-glycan, peptidoglycan, and lipopolysaccharide biosynthesis seemed to be well populated with the required enzymes. In addition, many of the required enzymes that convert L-amino acids to D-amino acids were identified. The portions of the pentose phosphate pathway allowing synthesis of the required precursors of nucleotide biosynthesis were also present. Purine and pyrimidine biosynthesis pathways were represented by a number of enzymes, permitting synthesis of several (but not all) bases and nucleotides. Interestingly, both strains appear to lack two enzymes that are essential for glycolysis; phosphofructokinase, and fructose-bisphosphate aldolase. If the enzymes are truly absent, this deficiency could be partially compensated by the pentose phosphate pathway. Another possibility is that *G. vaginalis *performs these functions using novel enzymes that were not identified in our similarity searches.

### Putative virulence factors in both strains

The only characterized virulence factor in *G. vaginalis *is vaginolysin [12]. Vaginolysin is a cholesterol-dependent cytolysin that may contribute to virulence by making cellular contents more available as a substrate for bacterial growth. This cytolytic action may also have the coincidental effect of making the vaginal epithelium more permeable to HIV virions. The gene for vaginolysin, *vly*, in strain 5-1 exhibited 89% identity at the nucleotide level and 94% identity at the amino acid level to *vly *from *G. vaginalis *strains 14019, 14018, and 49145 [12]. Vly from strains 5-1 and AMD were 99% identical at the amino acid level, differing at a single amino acid (T35A).

**Figure 1:**
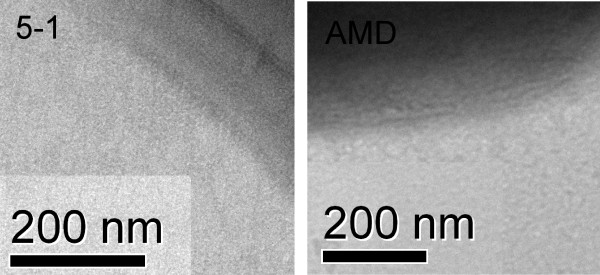
**Pili were not detectable on strains AMD and 5-1**. Tandem Electron Microsopy was used to investigate pilus expression by the two strains. The cell surfaces are shown at high resolution and suggest the absence of pilus production under the conditions used in this study.

Analysis of the *G. vaginalis *genome revealed a number of additional putative virulence factors, including a gene with similarity to the P30/P32 adhesins (GV51_0007, GVAMD_0260). These adhesins are produced by species of *Mycoplasma *and are involved in adherence to human tissues [22]. Previous reports have demonstrated that fresh clinical isolates of *G. vaginalis *are piliated, but suggest that pilus production may be lost upon *in vitro *passage of the bacteria [23,24]. However, even though loci likely to be involved in pilus assembly were present (GV51_0383 - 0387, GVAMD_1118 - 1122), we were unable to identify pili by transmission electron microscopy of strains 5-1 and AMD, even when the bacteria when *in vitro *culture was restricted (Fig. 1).

### Proteins encoded in only one strain

OrthoMCL analysis was performed to identify proteins putatively present in only one strain (Table 1). A number of these were small, hypothetical proteins that could represent pseudogenes and several did not exhibit significant similarity to any proteins of known function. Strain 5-1 contained a number of putative phage proteins that were absent in AMD. Strain AMD contained a number of putative serine-threonine kinases not present in 5-1, however the role of these kinases was unclear from similarity searches. AMD also contained an apparent toxin-antitoxin gene cassette. Interestingly, the antitoxin protein exhibited 100% identity to a protein from *A. vaginae *and 65% identity to a protein from *M. mulieris *suggesting that horizontal gene transfer occurs between BV-associated bacterial species.

**Table 1:** ORFs unique to one or the other *G. vaginalis *strains.

Unique to strain	Gene Identifier	Predicted function/homologues	Unique to strain	Gene Identifier	Predicted function/homologues
**AMD**	Gv1_0004 GVAMD_0044	**No significant similarity**	**5-1**	Gv1_0006 GV51_0275	**Hypothetical protein**

	Gv1_0004 GVAMD_0852	**No significant similarity**		Gv1_0006 GV51_0972	**Hypothetical protein**

	Gv1_0004 GVAMD_1243	**Hypothetical protein**		Gv1_0006 GV51_1019	**Hypothetical protein**

	Gv1_0004 GVAMD_0114	**No significant similarity**		Gv1_0008 GV51_0361	**Site specific recombinase**

	Gv1_0005 GVAMD_0177	**Serine/threonine protein kinase**		Gv1_0008 GV51_1208	**Phage-like integrase**

	Gv1_0005 GVAMD_1234	**Serine/threonine protein kinase**		Gv1_0009 GV51_0427	**Conserved hypothetical protein**

	Gv1_0005 GVAMD_1232	**Serine/threonince protein kinase**		Gv1_0009 GV51_1210	**Conserved hypothetical protein**

	Gv1_0005 GVAMD_0389	**Serine/threonince protein kinase**		Gv1_0010 GV51_0968	**Phage associated protein**

	Gv1_0011 GVAMD_0742	**ABC transporter**		Gv1_0010 GV51_0974	**Conserved hypothetical protein**

	Gv1_0011 GVAMD_0743	**ABC transporter**		Gv1_0012 GV51_0058	**Type 1 restriction modification system**

	Gv1_0016 GVAMD_0086	**No significant similarity**		Gv1_0012 GV51_0345	**Type 1 restriction modification system**

	Gv1_0016 GVAMD_0521	**No significant similarity**		Gv1_0013 GV51_0141	**Conserved hypothetical protein**

	Gv1_0017 GVAMD_0113	**Hypothetical protein**		Gv1_0013 GV51_0412	**Cell wall hydrolase**

	Gv1_0017 GVAMD_0456	**Hypothetical protein**		Gv1_0014 GV51_0363	**Cell wall protein**

	Gv1_0018 GVAMD_0143	**Conserved hypothetical, putative anti-toxin cassette**		Gv1_0014 GV51_1225	**Hypothetical protein**

	Gv1_0018 GVAMD_0681	**Conserved hypothetical**		Gv1_0015 GV51_1339	**Conserved hypothetical protein**

	Gv1_0019 GVAMD_0390	**2-component regulator**		Gv1_0015 GV51_1340	**Conserved hypothetical protein**

	Gv1_0019 GVAMD_0535	**Hypothetical, beta-lactamase class A**			

	Gv1_0020 GVAMD_0448	**Hypothetical protein**			

	Gv1_0020 GVAMD_0928	**Conserved hypothetical protein**			

	Gv1_0021 GVAMD_0931	**Hypothetical protein**			

	Gv1_0021 GVAMD_1004	**Hypothetical protein**			

### Putative antibiotic resistance determinants

BV can be difficult to treat and the rate of relapse or recurrence is 50% within a year [25]. Resistance to metronidazole, the drug of choice for BV therapy, has been seen in some strains of *G. vaginalis*, as has resistance to tetracycline [26]. We tested strains 5-1 and AMD for in vitro susceptibilities to 13 different antibiotics (Table 2). Both strains were sensitive to tetracycline (MIC = 0.5 μg/mL) and appeared to lack the *tetM *gene, found in tetracycline-resistant strains of *G. vaginalis *[27].

**Table 2:** In vitro susceptibilities of *G. vaginalis *strains to 12 antimicobial agents.

Antibiotic	MIC (μg/mL)	
	**Strain 5-1**	**Strain AMD**

Ampicillin	0.125	0.125

Nafcillin	0.12	0.12

Ciprofloxacin	1	2

Nalidixic acid	390	390

Chloramphenicol	0.5	1

Minocycline	0.156	0.078

Tetracycline	0.5	0.5

Erythromycin	0.032	0.128

Clindamycin	0.625	0.625

Kanamycin	32	32

Rifampin	0.063	0.063

Metronidazole	19.5	19.5

Therapeutically achievable levels of metronidazole are generally effective against bacteria with an MIC < = 8 μg/mL, whereas those with an MIC = 16 μg/mL are considered intermediate resistant and bacteria with an MIC > = 32 μg/mL are considered resistant [28]. Clinical cure rates of 75% have been reported for oral metronidazole therapy [29]. Both strains exhibited intermediate resistance with MICs of 19.5 μg metronidazole/ml. The most well-characterized mechanism of resistance to metronidazole is inactivation or deletion of genes with nitroreductase activity [30]. Both strains had nitroreductase genes (GV51_0575, GV51_0595, GVAMD_0064, and GVAMD_0125) but the expression of the genes and functionality of the proteins was not determined in this study.

Nalidixic acid resistance in *G. vaginalis *has been well established and strains 5-1 and AMD both exhibited resistance with an MIC above 300 μg/ml. The ORFs GV51_0431 and GVAMD_1169, which are 98% identical, are predicted to encode a protein with similarity to multidrug efflux pumps in the MATE (multidrug and toxin exclusion) family. Members of this family include *Staphylococcus aureus *MepA, which has been shown to confer fluoroquinolone resistance so this may be involved in resistance to nalidixic acid (18).

The strains also exhibited intermediate resistance to kanamycin. Both genomes contained a gene for a phosphotransferase enzyme family (GV51_0090, GVAMD_0184; 95% identical). This family includes proteins that inactivate certain aminoglycosides such as kanamycin. Therefore it is possible that the product of this gene plays a role in permitting kanamycin resistance in *G. vaginalis*.

The genomes also encoded an EmrB/QacA family protein (GV51_1060, GVAMD_0885; 99% identical), which could confer resistance to certain streptogramins, however, the strains were both susceptible to clindamycin and erythromycin, although strain 5-1 was more sensitive to erythromycin than was strain AMD.

### Analysis of surface structures

**Figure 2:**
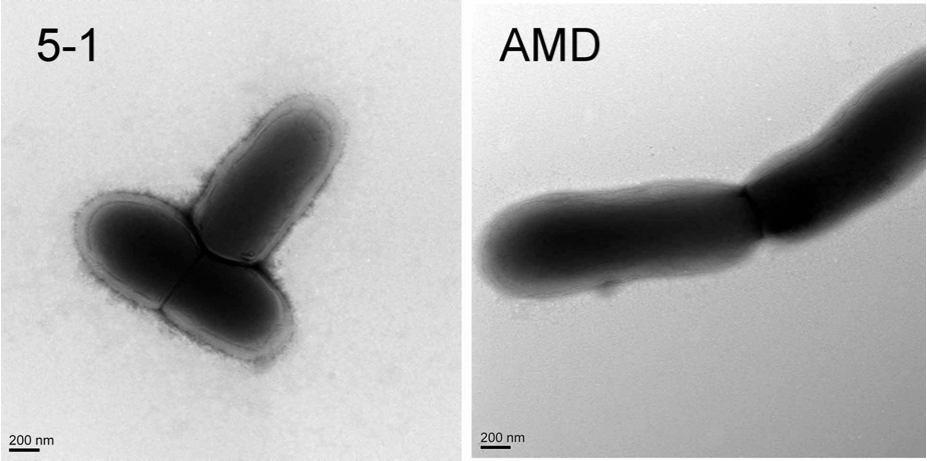
**Transmission Electron Microscopy of *G. vaginalis *strains**. The left panel is 5-1 while the panel on the right is AMD. When the strains were grown in the presence of 10% human serum, a capsule-like material was present on 5-1, but was undetectable on AMD.

Because we had noted a difference in the level of aggregation and biofilm formation of the two strains when they were cultured in 10% human serum (sBHIs), we analyzed the surface of the bacteria by transmission electron microscopy. We observed a layer that resembled a polysaccharide capsule on the surface of strain 5-1 that was absent on AMD (Fig. 2).

### Cytotoxicity

**Figure 3:**
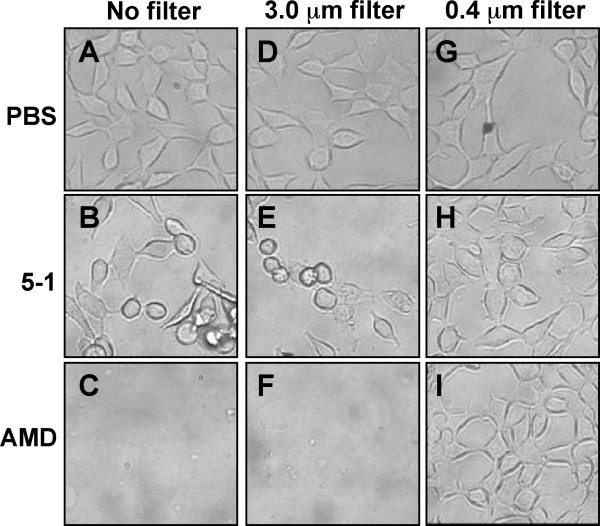
**Cytotoxicity caused by *G. vaginalis *strains**. Equal numbers of either AMD, 5-1, or PBS control were either added to a ME-180 monolayer directly (A-C), or were loaded into transwell filters with either 3 μm (D-F) or 0.4 μm (G-I) pore-size. The cells were monitored microscopically during the incubation for phenotypic alterations associated with toxic effects. After incubating the ME-180 cells with the bacteria for 3 hours, strain AMD had caused complete lysis of the monolayer whereas the cells incubated with strain 5-1 had just begun to exhibit rounding. The cytotoxic effects were completely blocked by the transwell with 0.4 μm pore size, which did not allow contact between the ME-180 s and the bacteria while the 3 μm pores, which did allow contact, permitted the cytotoxic effects.

Despite the finding that the *vly *genes of AMD and 5-1 encode nearly identical proteins, with only a single amino acid mismatch, cytotoxicity assays indicated that after 1 hour AMD, the BV-isolate, induced rounding and other cytopathologic changes in a monolayer of ME-180 cervical epithelial cells (data not shown), and complete lysis of the monolayer by 3 hours (Fig. 3B). In contrast, ME-180 cells exposed to the non-BV isolate, 5-1, were still attached and exhibited normal spreading epithelial morphology after 1 hour (data not shown) and had only begun to exhibit rounding and other cytopathogenic changes by 3 hours (Fig. 3C). Strain 5-1 led to complete disruption of the ME-180 monolayer after 12 hours (data not shown) suggesting that Vly from this strain was functional. To determine if differences in expression of *vly *could account for the reduced cytotoxicity associated with the commensal strain, we compared the *vly *promoter regions of the two strains and found that 5-1 was lacking 2 bp that were present in the AMD *vly *promoter. However, quantitative realtime RT-PCR indicated that transcriptional expression of the *vly *gene was equivalent in the two strains (data not shown).

### Adherence to epithelial cells

**Figure 4:**
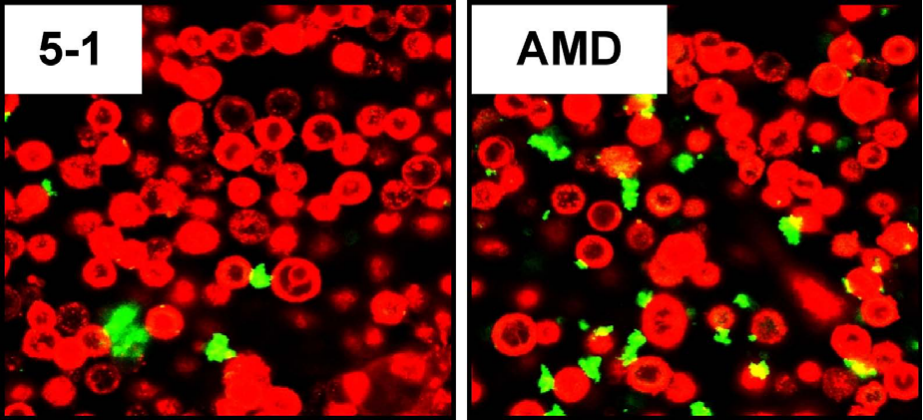
**Adherence of *G. vaginalis *to cultured vaginal epithelium**. Equal amounts of the indicated strains of *G. vaginalis *(green) were added to ME-180 cells (red). The cells were stained with BacLight green and Vybrant Red stains respectively. Adherence was analyzed by confocal microscopy following incubation and extensive washing with 1X PBS.

**Figure 5:**
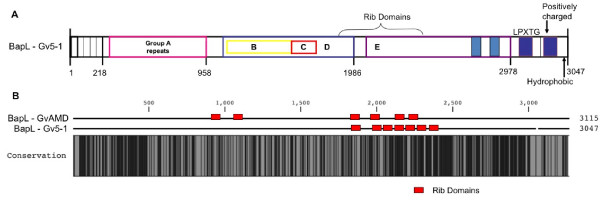
**BAP domain structure and inter-strain conservation**. A) Diagram representing the repeat structures found within the BAP encoded by *G. vaginalis *strain 5-1. Repeats units are diagramed above and named based upon the similarity to Bap proteins from other species. B) A ClustalW alignment of BAPs from strains AMD and 5-1 is shown, black represents 100% conservation. Rib domains are indicated by red boxes.

We analyzed the ability of the two *G. vaginalis *strains to adhere to cultured ME-180 cervical epithelial cells was assayed by confocal microscopy. While equal amounts of the two strains were added to ME-180 monolayers, adherence of strain AMD was much more pronounced relative to that of the non-BV isolated strain 5-1 (Fig. 4). It was also observed that strain AMD was more aggregative than was strain 5-1. This suggests that the capability of BV isolates to bind to and adhere to vaginal epithelium may be higher than non-BV isolates. As pilus expression could not be demonstrated in either strain, we searched for other putative adhesins. Both strains harbored a biofilm associated protein (BAP) family gene (Fig. 5A). BAP homologues in other species are involved not only in biofilm formation, but in adherence to epithelial cells as well, so we compared the sequences of the BAP genes encoded by 5-1 and AMD. ClustalW alignment of the amino acid sequences of the BAP family protein encoded by AMD and 5-1 showed considerable sequence differences between the two proteins (Fig. 5B). The BAP homologues had a highly repetitive structure as is characteristic of the other BAP family proteins (Fig. 5A) and a majority of the sequence differences between the two strains were located within the region containing the B, C, and D repeats. BAP is also predicted to contain several Rib domains. Rib domains were named after the Rib protein of Group B *Streptococcus*, an immunogenic surface proteins with a highly repetitive structure [31]. The number and distribution of Rib domains in the BAP homologues differed between AMD and 5-1.

### Role of adherence in cytotoxicity

Other cholesterol-dependent cytolysins depend upon adherence to exert their activity [32]. Because we observed that AMD exhibited increased adherence, we investigated the possibility that this was related to the greater cytotoxic activity associated with AMD. *G. vaginalis *was added to transwell filters with a pore-size equal to either 3 μm, to allow the bacteria to penetrate the filter and make contact with the monolayer, or 0.4 μm, to prevent direct contact of the bacteria and monolayer but to allow proteins to flow through to the ME-180 s. We found that direct contact with the monolayer was required for cytotoxic activity (Fig. 3D-I), suggesting that the disparity in the adherence of the two strains could account for differences in cytotoxic activity.

### Capacities for biofilm-formation

**Figure 6:**
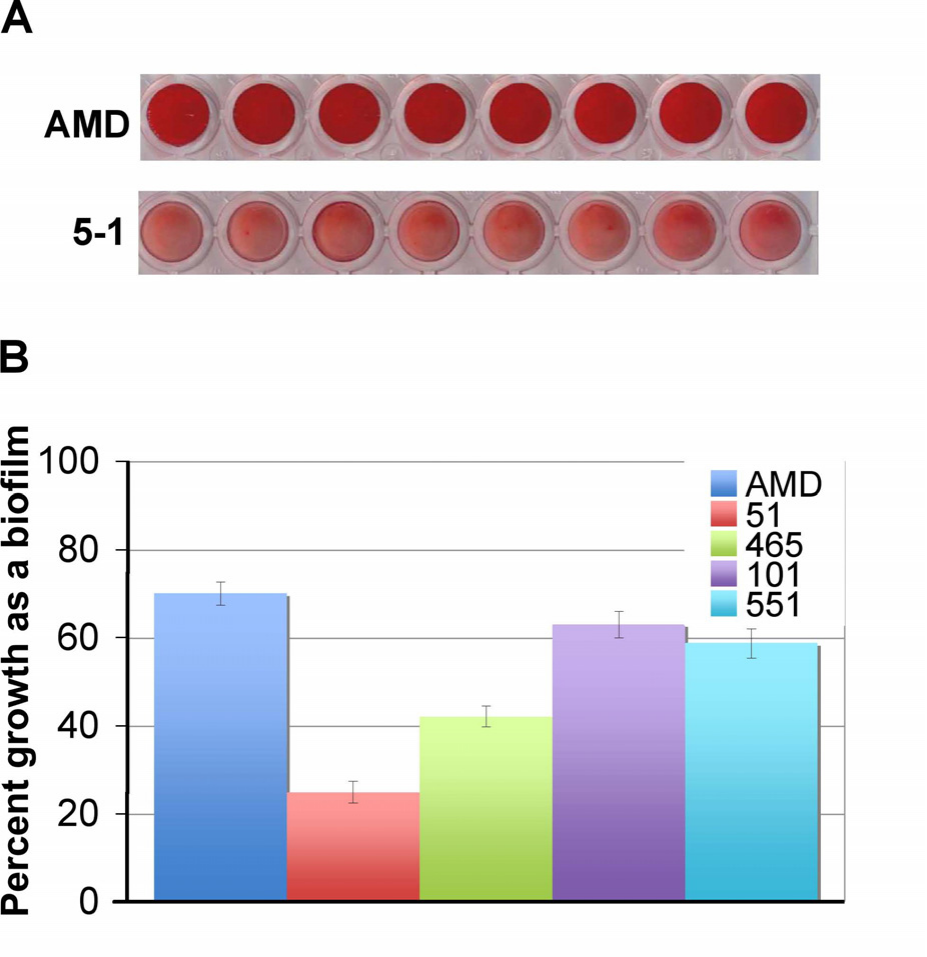
**Biofilm-forming capacity of *G. vaginalis *isolates**. A) Biofilms were stained with safranin for a visual assessment of biofilm thickness. B) Because the BV-associated isolate AMD exhibited significantly greater biofilm-forming capacity than the non-BV isolate 5-1 we tested an additional non-BV isolate (465) and two additional BV isolates (101 and 551). The percent growth as a biofilm was calculated as OD_595 _biofilm/(OD_595 _biofilm + OD_595 _planktonic) and is represented graphically. The error bars represent standard deviation of 8 independent data points.

The ability to form a biofilm is a marker of potential virulence and a cause of recurrent infections. Since BAP proteins are known to play a role not only in adherence but in biofilm formation in other species [33], we chose to investigate whether the disparity in the sequences and expression of the BAP homologues could translate into differences in biofilm forming activity. Safranin staining of biofilms (Fig. 6A) produced by *G. vaginalis *strains AMD and 5-1 after 24 hours of growth showed that the BV-isolate AMD produced a thicker biofilm. Quantitative measurement of biofilm growth as percentage of total growth indicated that AMD had a significantly greater biofilm-forming capacity than the non-BV isolate 5-1 (Fig. 6B). The relationship between biofilm capacity and association with BV was further investigated using additional strains. We found that two other *G. vaginalis *BV-isolates, strains 101 and 551 produced similar levels of biofilm growth as did strain AMD and significantly more than strain 5-1. A second healthy isolate, strain 465, showed significantly less biofilm growth than the BV-isolates (Fig. 6B). These data suggest that BV-isolates tend to produce more biofilm growth than do non-BV associated isolates of *G. vaginalis*, which would reasonably be consistent with greater virulence.

## Discussion

*G. vaginalis *can be isolated from the majority of BV cases but it can also be isolated, albeit at much lower numbers, from healthy women. Very little is currently known about the genetic composition of *G. vaginalis*, the diversity of strains or about its physiology. It has been reported that certain biotypes of *G. vaginalis *are more frequently associated with BV [20]. In order to investigate the possibility that certain strains are more likely to be associated with BV, we sequenced a BV-associated strain and a healthy isolate and characterized their virulence potentials with a series of *in vitro *assays for cytotoxicity, adherence, and biofilm formation. We found that the BV-associated strain was significantly more cytotoxic than the non-BV isolate suggesting that differing levels of cytotoxicity may be related to the propensity of a strain to cause disease. Proteins encoded by the *vly *genes differed by only a single amino acid and the promoters differed by only 2 bp. The disparity in the level of cytotoxicity of the two strains could be related to the amino acid substitution, but the residue is not near the active site or other amino acids known to play a key role in function [12]. The differences in cytotoxicity are likely a function of adherence. We found that strain AMD adheres more avidly to cultured vaginal epithelial cells than does the non-BV isolate, 5-1. Therefore it is possible that the increase in cytotoxicity seen with strain AMD is simply due to its ability to adhere and thereby deliver vaginolysin more directly to the eukaryotic monolayer. Consistent with this hypothesis was the finding that a 0.4 μM filter effectively blocked *G. vaginalis*-mediated cytotoxicity. If the toxic effector molecule produced by the bacteria was freely secreted in sufficient concentrations and the secreted form was cytotoxic, then phenotypic changes of the ME-180 monlayer would have been seen even under conditions that prevented direct cell to cell contact, i.e. the 0.4 μM filter. However the filter ablated cytotoxicity in ME-180 s suggesting that direct contact between *G. vaginalis *and the vaginal epithelium is required for effective delivery of the *vly *gene product, as is the case with other cholesterol-dependent cytolysins [32].

These results have also demonstrated a clear difference in the ability of various strains to adhere to cultured cervical epithelial cells. While both AMD and 5-1 were able to adhere to the ME-180 monolayer, AMD exhibited heightened adherence. Adherence is a prerequisite for infection, and these results suggest that BV isolates are able to adhere, and thus establish an infection much more readily than non-BV isolates. Factors influencing the ability of a given strain to adhere to eukaryotic cells would be governed by the proteins and structures present upon the bacterial cell surface and the interaction between those factors and the ME-180 surface proteins and structures. We found a gene encoding a biofilm associated protein (BAP) family protein. BAP proteins are large, cell wall-anchored adhesins that can mediate both adherence to host cells and intercellular adherence, which contributes to biofilm formation [33,34]. Interestingly, the gene sequences for AMD and 5-1 BAP were quite disparate. This was particularly noticeable in the repeat regions, the region of BAP proteins that generally mediates adherence. We also noted that the healthy isolate appeared to be coated in a capsular structure, whereas the BV-associated isolated did not appear to express this structure. This may negatively impact adherence to vaginal epithelial cells, or biofilm formation. Pneumococci produce a capsule that has been linked to a decrease in biofilm formation supporting this hypothesis [35]. Both genomes contained multiple potential operons for capsular polysaccharide biosynthesis although there was significant divergence in the proteins encoded by the two strains.

We also found a significant difference in the propensity of the two strains to form a biofilm *in vitro*. Biofilm formation by *G. vaginalis *has recently been implicated in BV [11]. The ability for a strain to grow as a biofilm would likely confer resistance to mucosal immune defenses and antibiotic resistance, which could contribute to initial and recurrent colonization. Furthermore, lactobacilli normally associated with the healthy vagina produce byproducts such as lactic acid and hydrogen peroxide that normally suppress the growth of anaerobes such as *G. vaginalis*, but biofilm formation leads to increased resistance to these byproducts [36]. Therefore, biofilm formation may enable proliferation of *G. vaginalis *even in the presence of lactobacilli. Finally, biofilm formation is associated with increased antibiotic resistance and appears to play a role in treatment failure and recurrence in cases of BV [37]. The difference in biofilm forming capacity between these two strains could be related to the differences between the respectively encoded BAPs. The differences in domain distribution within the central repeat region and, potentially more importantly, the number and distribution of the Rib domains and repeats in strain AMD could better promote adherence and/or bacterial aggregation. Furthermore, our results were suggestive of a correlation between greater biofilm-forming capacity and association with BV.

This work clearly demonstrates strain differences between *G. vaginalis *isolates that could impact the ability of this organism to cause disease. The precise role for *G. vaginalis *in BV pathogenesis is still unclear, but this study suggests an explanation for the presence of this organism in the absence of BV. A limitation of this study is the restricted number of strains studied. Advances in sequencing technology and the Human Vaginal Microbiome Projects, taking place at Virginia Commonwealth University and the University of Maryland, will lead to the analysis of additional strains, which will reveal further insight into the role of the genetic background of *G. vaginalis *strains in pathogenesis and to clearly demarcate the differences between strains that are directly related to the propensity to cause disease.

## Conclusions

This study provides evidence to support the hypothesis that certain biotypes of *G. vaginalis *are unable or unlikely to cause disease while other strains are better suited to elicit disease. The results suggest that the strains that fail to elicit BV may not be able to adhere to, or form a biofilm on the vaginal epithelium as avidly as strains that cause BV. Results from genomic sequence analysis identified significant differences between a strain associated with BV and a strain from a healthy subject. The sequencing data reveals genes that may be involved in virulence and opens up avenues for further study of the pathogenesis of BV.

## Methods

### *Gardnerella *strains and growth conditions

*Gardnerella vaginalis *strains 5-1 and 465 were originally isolated from two healthy women without BV as diagnosed by the Nugent gram stain scoring system and strains 101 and 551 were isolated from women diagnosed with BV at Brigham and Women's Hospital, Boston, MA [38]. Strain AMD was isolated from a woman diagnosed with BV based on Amsel criteria at VCU Women's Health Clinic [39]. The strains were grown in Brain Heart Infusion (BHI) broth (EMD, Gibbstown, NJ) at 37°C using the AnaeroPack system (Mitsubishi Gas Chemical Co, Tokyo, Japan).

### DNA isolation

*G. vaginalis *strains were grown in 200 mL BHI overnight. The cells were collected by centrifugation, resuspended in 7 mL TNE buffer (10 mM Tris HCl pH 8.0, 10 mM NaCl, 10 mM EDTA) containing 1% Triton X-100, and 10 mg lysozyme/mL, and incubated at 37°C for 30 minutes. Proteinase K (0.3 mg/mL) was added and the suspension was incubated for 1 hour at 65°C. DNA was extracted with 7 mL 1:1 phenol/chloroform, followed by 7 mL chloroform, and precipitated with 1/10 volume 3 M sodium acetate and 2 volumes ethanol.

### Sequencing

Sequencing was performed using Roche 454 technology. One full run of GS FLX and one half run GS FLX XLR were done for strain 5-1 (coverage of ~175 X), while one half run of GS FLX XLR was done for strain AMD (~130 × coverage). Five micrograms of each strain's DNA was sequenced as per the standard Roche sequencing protocol.

### Sequence analysis

Genomes were assembled by Roche's software Newbler 2.0.00.20 using default parameters. Resulting contigs were analyzed by Glimmer 3 [40] for gene calling. Transfer RNA genes were predicted using tRNAscan-SE 1.23 [41] and ribosomal RNA genes were found by similarity searches. Sequences were initially annotated by comparison with currently annotated bacterial sequences present in NCBI's NR protein database. Metabolic reconstruction and Gene Ontology classification assignments were performed using ASGARD [42], using the UniRef100 database [43]. Other annotation features were predicted using several programs, namely: transmembrane domain, by TMHMM 2.0c [44]; signal peptide, by SignalP 3.0b [45]; protein secretion probability, by SecretomeP 2.0 [46]; COG similarities and Pfam domain composition, by rpsblast [47]; protein characteristics (isoelectric point, molecular weight, and charge) by pepstat [48]. The resulting annotation and sequence assemblies were uploaded to GBrowse [49] installations for visualization and analysis. Overall DNA sequence identity comparison of the two strains was performed using MUMmer 3.20 [50]. Determination of putative ortholog relationship (and therefore the determination of gene uniqueness) between the genes of the two strains was performed by OrthoMCL 2.0b6 [51].

### Antibiotic resistance assay

*G. vaginalis *strains 5-1 and AMD were diluted to an OD_600 _= 0.1 in BHI supplemented with 1% yeast extract, 2% gelatin, and 0.1% starch (sBHI) containing 1% glucose (sBHIg). Antibiotics were serially diluted 2-fold in 200 μL sBHIg in mictotiter wells and 3 μL of the bacterial suspension was added to each well. The microtiter plates were incubated anaerobically for 24 hours and the lowest concentrations of antibiotics that prevented visible bacterial growth were recorded.

### Electron Microscopy

Bacteria grown overnight in sBHI supplemented with 10% human serum (sBHIs) were collected by centrifugation, washed in sterile deionized water, spotted onto formvar-coated 200-mesh copper grids (Electron Microscopy Sciences, Hatfield, PA), stained with 2% phosphotungstic acid, and analyzed using a Jeol JEM-1230 transmission electron microscope equipped with a Gatan UltraScan 4000SP 4K x4K CCD camera.

### Cytotoxicity

Strains AMD and 5-1 were cultured in sBHIs, collected by centrifugation, and resuspended in PBS to an OD_600 _= 0.15. ME-180 monolayers were cultured in 96 well plates to ~90% confluence, the media was replaced with 100 μL 1× PBS, and 100 μL of the bacterial suspensions was added. To asses the contact dependence of *G. vaginalis*-associated cytotoxicity, ME-180 monolayers were cultured in 6 well plates, the media was replaced with 2 ml of 1× PBS, a sterile transwell with pores of either 0.45 μM or 8 μM was inserted into a well, and 2 ml of the bacterial suspension was added to the top portion of the transwell. The monolayers were monitored every hour by light microscopy for cytopathogenic changes, such as cell rounding, loss of adhesion, and disruption of the monolayer. Photos were taken using an Olympus CK2 microscope at magnifications of 100× and 400×.

### RNA Isolation and Quantitative RT-PCR

Overnight cultures of the *G. vaginalis *strains were subcultured 1:20 in 5 ml of sBHIs and grown for ~6 hours anaerobically at 37°C to mid-exponential phase. The cells were collected and resuspended in 500 μl of buffer RLT (Qiagen, Germantown, MD). The suspension was transferred to Lysing Matrix B tubes (MP Biomedicals, Solon, OH) and 500 μl of a 5:1 mixture of acid phenol:cholorform (Ambion, Austin, TX) was added. The cells were lysed using a FastPrep FP120 Instrument (Thermo Scientific, Waltham, MA) with settings of power 6 and duration of 40 seconds. Samples were centrifuged 5 minutes 20,817 rpm at 4°C, the upper layer was transferred to a new microfuge tube containing 500 μL EtOH (500 μl), the samples were mixed by inversion, and the RNA purification was performed using the RNeasy kit (Qiagen) according to manufacturer's instructions, and the RNA was eluted in 90 μl of RNase-free water. DNA was removed using the Turbo DNA-free kit (Ambion). RNA was quantified by measurement of absorbance at 260 and 280 nm. Synthesis of cDNA from 1 μg RNA was carried out using the Tetro® cDNA synthesis kit (Bioline, Taunton, MA) and 1 μM Gv16sc-REV: 5'AGGTACACTCACCCGAAAGC3', MH8: 5'GTTAATGGTGCGCGATTTGC 3', and MH6: 5'GTTGTTAAAGAACACATCGAAG3'. Resulting cDNA was diluted 1:100, and 2 μl of the diluted cDNA was used as a template for realtime RT-PCR in reactions that included 0.4 μM of each forward and reverse primer as well as Sensimix Plus + Fluorescein (Quantace, Norwood, MA) at a final concentration of 1×. Primer sets were as follows: 16 S - Gv16sc-FWD: 5'CACATTGGGACTGAGATACGG3' and Gv16sc-REV; *vly *- MH7: 5'CTTGCGCAGCCAGCAAGG3' and MH8; *bapL *- MH5: 5'GTGTCATTGAGCACACTTGC3' and MH6. Control reactions lacked reverse transcriptase enzyme to ensure that DNA contamination was minimal. Reactions were performed on an IQ5 Multicolor Realtime PCR Detection System (Bio-Rad, Hercules, CA). Optimal hybridization temperatures were determined by performing reactions over a 10°C gradient. Primer efficiencies were determined by using five, 5-fold dilutions of cDNA as template and the resulting Ct values were plotted versus the Log10 of the dilution factor, yielding a straight line, the slope of which was then used in the following equation: Efficiency = 10 (1/m) where m = slope. Reactions were incubated for 10 minutes at 95°C then cycled for 35 rounds of 10 seconds at 95°C, 10 seconds at the determined hybridization temperature, and then 15 seconds at 72°C during which data, SYBR green fluorescence, was collected. Data following RT-PCR was corrected for primer efficiency by applying the formula: Corrected = EfficiencyCt. The corrected value obtained was normalized to 16 s rRNA expression. Averages were obtained from technical replicates and standard deviation was determined. Biological triplicates were performed for each sample.

### Adherence

Bacteria were grown in sBHIs, and ~10^8 ^were collected by centrifugation and resuspended in 1× PBS. ME-180 cervical epithelial cells (ATCC) were cultured at 37°C in 5% CO_2 _in McCoy's 5A medium (Quality Biologic, Gaithersburg, MD) supplemented with 10% fetal bovine serum and 1IU ml^-1 ^penicillin/streptomycin (MediaTech, Manassass, VA) in 6-well polystyrene plates (Greiner, Monroe, NC) to 90% confluence. ME-180 cells were stained with 2.5 μM Vybrant red membrane stain in PBS and bacteria were stained with a solution of 0.5 μM BacLight green (Invitrogen, Carlsbad CA) in PBS at 37°C for 60 minutes. Following staining, epithelial cells and bacteria were washed twice in 1× PBS, the bacteria were added to the monolayer in a final volume of 3 mL PBS, and the plates were centrifuged to maximize contact between the bacteria and epithelial cells. The bacteria were allowed to adhere at 4°C for 15 minutes, the monolayer was washed 3 times with 1× PBS, and adherence was visualized using a Zeiss LSM 510 META NLO multi-photon laser scanning microscope with an Achroplan 63× water dipping objective. BacLight green was excited with a 488 nm argon laser, Vybrant Red with a 633 nM laser and emissions filters 515 nm (green) and 630 nm (red) were used.

### Biofilm formation

Strains 5-1 and AMD were grown in sBHIs in 96 well tissue culture-treated plates (Greiner) anaerobically at 37°C for 24 hours. For a qualitative assessment of biofilm formation, non-adherent bacteria were removed from the wells and adherent bacteria were stained with safranin. For a quantitative measure of biofilm formation, planktonic bacteria were removed from the wells and transferred to new wells, and the biofilms were resuspended in fresh media. The OD_595 _was determined for both planktonic as well as biofilm growth. The percent growth as a biofilm was calculated as OD_595 _biofilm/(OD_595 _biofilm + OD_595 _planktonic).

## Authors' contributions

M.D.H. performed cytotoxicity, adhesion, biofilm, MIC, and qRT-PCR studies, performed electron microscopic analysis and wrote the paper. J.M.A. performed sequencing of G. vaginalis strains, subsequent anaylsis of results, and wrote the paper. G.A.B. contributed analytical tools required for completion of DNA sequencing and analyzed sequencing data. J.F.S. participated in the experimental design of the study and edited the manuscript. J.L.P. performed MIC, protein alignment, cytotoxicity, and qRT-PCR studies. A.T.O. performed cytotoxicity studies. P.H.G. contributed to research design and participated in editing of the manuscript. K.K.J. designed the research contained in this paper, analyzed and interpreted data, and edited the manuscript. All authors read and approved the final version of the manuscript.

## Competing interests

The authors declare that they have no competing interests.
